# Association Analyses of Physical Fitness Parameters and Anxiety Symptoms in Chinese College Students

**DOI:** 10.3390/ijerph20010623

**Published:** 2022-12-29

**Authors:** Jianjun Yin, Lingfeng Kong, Yufei Cui

**Affiliations:** 1 Department of Physical Education, Guangdong University of Finance and Economics, Guangzhou 510320, China; 2 Department of Physical Education, Hohai University, 1 Xikang Road, Nanjing 210098, China; 3 Department of Physical Education, Huaiyin Institute of Technology, Huai’an 223003, China

**Keywords:** physical fitness, anxiety, college students, epidemiological study

## Abstract

Poor physical fitness is related to many negative health outcomes, including mental disorders. However, the relationship between physical fitness and anxiety symptoms among college students remains unclear. Therefore, this study investigated whether physical fitness is related to anxiety symptoms in Chinese college students. Cross-sectional data were collected from 6635 men and 4482 women. Physical fitness was measured via a 50-m sprint, a sit-and-reach test, vital capacity, and a standing long jump for both sexes; 1000-m run and pull-up tests for males; and 800-m run and sit-up tests for females. The seven-item Generalized Anxiety Disorder Scale (GAD-7) was used to evaluate anxiety symptoms. Multivariate linear regression showed that better physical fitness was related to lower GAD-7 scores. In addition, multivariate logistic regression analysis showed that better ability in the 50-m sprint, sit-and-reach test, pull-up test, and vital capacity was related with a lower risk of anxiety symptoms in males, and better ability in the 800-m run and standing long jump was related with a lower risk of anxiety symptoms in females. In conclusion, physical fitness was inversely associated with anxiety symptoms in male and female college students. This association was also independent of confounding factors.

## 1. Introduction

Mental disorders among young adults are a public health issue worldwide. Anxiety symptoms are widely known as a mental disorder and have also been proven to be a risk factor for many diseases, such as high blood pressure [1], diabetes [2], osteoporosis [3], and sleep problems [4], and are also associated with a reduced quality of life [5]. The prevalence of anxiety symptoms among university students is higher than that of the general population [6]. Some studies have claimed that the prevalence of anxiety among university students exceeds 50% [6,7,8]. With university being the first step towards independent life, students have to adequately manage their relationships with classmates and roommates on top of their busy academic schedules, and Chinese university students have faced severe employment pressure in recent years. As a result, an anxious mood may be an inevitable part of their daily lives. Young adulthood is particularly important, as this phase bridges adolescence and adulthood. Properly handling anxiety symptoms and developing a healthy mental state during this phase can have positive repercussions for the rest of their lives.

As an important indicator of health, physical fitness is also considered a predictor of mental illness [9]. However, recent data has shown that the physical fitness of Chinese adults, such as strength, flexibility, and balance, has declined over the past decade [10]. This has caught the attention of the Chinese government, resulting in the introduction of many relevant policies to improve the physical fitness of Chinese people. Physical fitness has also been reported to be related to cardiovascular disease risk [9] and bone health [11], and serves as a strong predictor of mortality [12] and physical disability in the next 25 years [13]. Recent studies have also discussed the association of physical fitness with mental health. Overall, most of these studies have considered depressive symptoms as the outcomes. Given that better physical fitness is related to a better state of health and mood, it is reasonable to assume that physical fitness is also associated with anxiety symptoms. Although several previous studies have verified the relationship between physical fitness and anxiety symptoms, these studies have been limited to children, adults, older adults, and patients, with little known about this association in the young adult population. Furthermore, these studies have differed in their evaluation of physical fitness—some studies have focused on the association between grip strength and anxiety symptoms [14,15], whereas others have investigated the association between cardiovascular fitness [16,17], balance, and mobility and anxiety symptoms [18]. Therefore, this cross-sectional study was designed to examine the association between physical fitness (running ability, muscle strength, flexibility, and vital capacity) and anxiety symptoms among Chinese college students. Based on previous research findings, we hypothesized that college students’ physical fitness was negatively associated with anxiety symptoms.

## 2. Materials and Methods

### 2.1. Participants

This study adopted a cross-sectional design, and participants were recruited from the Huaiyin Institute of Technology. Information about the characteristics and lifestyles of students who participated in annual physical examinations at the college in 2018 was collected. Prior to the physical examination, students completed questionnaires under the guidance of trained instructors. We excluded students with physical disabilities, cardiovascular disease, or respiratory disease, students who were unable to participate due to special circumstances before the physical examination, as well as students with missing data from the analysis (*n* = 1463). The final 11,117 students (6635 males and 4482 females) were analyzed in the study. The Ethics Committee of the Huaiyin Institute of Technology approved this study.

### 2.2. Physical Fitness

Physical fitness data were obtained from annual student physical health examinations, which are conducted based on the National Student Physical Health Standard (revised in 2014). The standard is intended to establish and improve the physical health monitoring and evaluation system for the national student population and encourage students to actively participate in physical exercise and improve the physical and mental health of young people [19]. The examinations were performed and recorded by experienced trained instructors at the sports facilities of the college. The physical fitness test consisted of eight items: a 50-m sprint (recorded in seconds), a sit-and-reach test (recorded in cm), a standing long jump (recorded in cm), and vital capacity (recorded as mL/body weight (kg)) for both sexes; a pull-up (recorded in times) and 1000-m run (recorded in seconds) for males; a 800-m run (recorded in seconds) and a sit-up test (recorded in times) for females. The criteria for university students were used in all physical fitness tests, and warm-up exercises were performed before all physical fitness tests.

### 2.3. Anxiety Symptoms

Anxiety symptoms were evaluated using the seven-item Generalized Anxiety Disorder Scale (GAD-7), a self-reported questionnaire used to screen for anxiety symptoms in the general population. The seven items are rated on a four-point scale scored from 0 to 3, and a total score ranging from 0 to 21; the higher the score, the more severe the symptoms. The effectiveness of the GAD-7 has been validated in a previous study [20], and a cut-off value of ≥8 was used in this study to distinguish whether participants suffered from anxiety symptoms [21].

### 2.4. Covariates

Height was measured using a stadiometer, and weight was measured using a weight scale in the physical health examinations. Body mass index (BMI) was calculated as kilograms per meters squared. The basic information of the participants was obtained using a self-reported questionnaire. Grades were categorized as freshmen, sophomore, junior, or senior. Race was defined as a Han or minority race. Living status was classified into two categories: dormitory and other. Living expenses were categorized into tertiles: low, medium, or high. Cigarette smoking was defined as being a smoker or a non-smoker. Drinking status was defined as the frequency of alcohol drinking per week, and then divided into three categories: a nondrinker; a drink 1–2 times/week; or a drink > 2 times/week. Sleep duration was obtained by asking participants how long they slept at night, and the response was divided into two categories: 7–8 h or other. An International Physical Activity Questionnaire (IPAQ) was used to assess daily physical activity [22], and the metabolic equivalent of tasks (METs) in hours per week was calculated.

### 2.5. Statistical Analysis

Statistical analyses were performed using the Statistical Package for Social Sciences version 24.0 for Macintosh. Group comparisons were carried out using a *t*-test and χ^2^ test for continuous variables and categorical variables, respectively. The results for continuous and categorical variables in the basic characteristics were shown as mean values and 95% CI and percentages, respectively. The adjusted linear association between each physical fitness item and GAD-7 score was examined using multivariate linear regression analysis, β was calculated, and the adjusted association between physical fitness (category) and anxiety symptoms (yes or no) was examined using multivariate logistic regression analysis; odds and 95% CIs were presented as results. Confounding factors included grade, race, BMI, physical activity, living expenses, cigarette smoking, drinking habits, living status, and sleep duration. *p* < 0.05 was considered statistically significant. The test items of physical fitness for men and women were different; thus, we separately analyzed both sexes.

## 3. Results

This study included 6635 men and 4482 women. The mean GAD-7 score was 3.8 ± 3.6 and 3.9 ± 3.6 for males and females, respectively, and the anxiety symptoms proportion was 42.4% for men and 43.0% for women. The participants’ characteristics are shown according to their anxiety levels in Table 1. Among male participants, those who were freshmen and drinking 1–2 times per week had a higher proportion of anxiety symptoms. In contrast, male participants who were sophomores, juniors, seniors, and non-drinkers had a lower proportion of anxiety symptoms. In addition, participants without anxiety symptoms had higher physical ability in all items of physical fitness, except the 1000-m run. Among female participants, those who were freshmen and drinking > 2 times per week had a higher anxiety symptom proportion. In contrast, female participants who were sophomore, junior, senior, and 7–8-h sleepers had a lower proportion of anxiety symptoms. Moreover, participants without anxiety symptoms had higher physical ability in all items of physical fitness, except the sit-and-reach test.

Adjusted linear associations between physical fitness and GAD-7 scores are shown in Table 2. After adjusting for confounding factors, a high GAD-7 score was related to a slow 50-m sprint time, a high vital capacity, and a high score in the sit-and-reach test and standing long jump in male participants. In female participants, a high GAD-7 score was related to a low speed in the 800-m run and short distance in the standing long jump.

Table 3 shows the association of physical fitness with anxiety symptoms among male participants. In the adjusted model, compared with the fast category of the 50-m sprint, the odds ratios and 95% confidential intervals of the medium and slow categories were related to a higher proportion of anxiety symptoms (*p* for trend = 0.001). Compared with the short category of the sit-and-reach test, the odds ratios and 95% confidential intervals of the medium and long categories were related to a lower proportion of anxiety symptoms (*p* for trend = 0.006). Compared with the low category of pull-up, the odds ratios and 95% confidential intervals of the medium and high categories were related to a lower proportion of anxiety symptoms (*p* for trend = 0.011). Compared with the low category of vital capacity, the odds ratios and 95% confidential intervals of the medium and high categories were related to a lower proportion of anxiety symptoms (*p* for trend = 0.007).

Table 4 shows the association of physical fitness with anxiety symptoms among female participants. Compared with the fast category of the 800-m run, the odds ratios of the medium and slow categories were associated with a higher proportion of anxiety symptoms (*p* for trend = 0.012). Compared with the short category of standing long jump, the odds ratios and 95% confidential intervals of the medium and long categories were associated with a lower proportion of anxiety symptoms (*p* for trend = 0.021).

## 4. Discussion

This study was conducted to identify the association between physical fitness and anxiety symptoms in college students. The results indicated that better physical fitness, including running ability, flexibility, and muscle strength, was related with a lower proportion of anxiety symptoms. This relationship was present in both males and females and was independent of confounders. To the best our knowledge, this paper firstly demonstrated a relationship between physical fitness and anxiety symptoms among Chinese college students. Therefore, our findings strengthen research evidence in the field related to physical fitness and anxiety symptoms.

Many kinds of assessment for anxiety symptoms have been used in previous studies, such as GAD-2 [23], DAS-21 [8], and STAI [6]. However, in our study, we used GAD-7 to evaluate anxiety symptoms, and set a cut-off point of ≥ 8 for anxiety symptoms. Therefore, it may be difficult to know whether the proportion of anxiety symptoms in our study were higher or lower compared with previous studies. On the other hand, one of those studies used GAD-7 to evaluate anxiety symptoms in Hong Kong university students [7], but the cut-off point was set to ≥5. It showed that the proportion of anxiety symptoms was 54.4% in the population, which was close to the anxiety proportion in our study (42.7%); the anxiety proportion could be increased if we set the cut-off point to ≥5 in our study.

Some studies have investigated the relationship between physical fitness and anxiety symptoms. Yansong et al. studied 269 children aged 7–12 years in China and found that physical fitness, including the vital capacity of the lung, a 50-m sprint, sit-and-reach, rope-skipping, and sit-ups, were inversely associated with anxiety symptoms [24]. Verónica and Brett found that grip strength was inversely related to anxiety symptoms in a sample of 162,167 British adults aged 38–70 [25] and 3952 Irish older adults aged over 50 [14]. Ron’s study indicated that the capacity for 10-m walking, a unipedal stance test, and a timed up-and-go test were inversely associated with the prevalence of anxiety symptoms in healthy adults aged 18–65 [18]. In addition, another epidemiological study of 2088 adults aged 20–39 indicated that people with higher levels of cardiorespiratory fitness had lowered odds of generalized anxiety. Summarizing these previous studies, their findings are consistent with our study findings, which revealed an inverse association between physical fitness and anxiety symptoms. However, some differences also exist between the studies, such as different assessments of anxiety symptoms and samples from different countries. Moreover, as mentioned above, most previous studies only used a few fitness items to represent physical fitness, such as grip strength and cardiorespiratory fitness; however, in our study, we used more items for physical fitness, including a 1000-m and a 800-m run (stamina), a 50-m sprint and a standing long jump (lower limb strength), the pull-up and sit-up tests (upper limb strength), the sit-and-reach test (flexibility), and vital capacity. In addition, these studies examined this association in children, adults, older adults, and patients. However, the lifestyles and mental statuses of these populations are different from university students. Thus, our study made up for this lack, and proves this association in university students. In particular, Chinese students have physical education classes in high school. However, the class hours of physical education are reduced after entering university, and there are no physical education classes throughout the three years of university, which greatly reduces opportunities for physical exercise. As a result, physical fitness decreases. Better physical fitness could affect the psychological state, so as to eliminate stress and stabilize emotions, among others. Different from other ages, college age occurs during the final stage of adolescence and the period of transition to adulthood. Students need to prepare the psychosocial skills that will be beneficial in a stressful society in the future. Thus, it may be more important for them to maintain a better physical fitness. Several possible mechanisms for the relationship between physical fitness and anxiety symptoms could be considered. First, it has been reported that higher levels of physical fitness are related to a reduction in inflammatory levels [26], and inflammatory cytokines are higher in individuals with anxiety disorders [27]. Thus, it could be considered that higher physical fitness may contribute to a lower level of anxiety symptoms through this indirect link. Furthermore, people with higher physical fitness levels tend to have positive perceptions of anxiety symptoms [28] or positive moods, and so feel less anxious. On the other hand, higher physical fitness may also be associated with some positive factors in people’s daily lives, such as high physical activity, normal body weight, or good sleep, which consequently influence anxiety symptoms. However, we adjusted these factors as covariates for the relation of physical fitness with anxiety symptoms; the results showed that these factors were independent of the association in the present study.

The strength of this study is the relatively large sample size and that physical fitness was evaluated using eight test items. In addition, GAD-7 was used as a continuous and categorical variable. However, the present study faces the following limitations. First, as this is a cross-sectional study, we cannot prove a causal inference on whether better physical fitness leads to a lower risk of anxiety symptoms, or whether students with anxiety symptoms simply tend to be physically less fit. Second, the instructors recorded the time of the running test using a stopwatch. Although the instructors were trained before the tests, measurement bias may also exist between them. An electronic timing system should be considered for recording the time in future studies. Third, physical exercise may be a confounder or a mediator in the association between physical fitness and anxiety symptoms. However, we did not collect any information about physical exercise. Although we used physical activity to adjust the association, physical activity is not exactly the same as physical exercise. Fourth, while adjusting for some confounding factors for the relation between physical fitness and anxiety symptoms, we cannot eliminate the possibility of other factors that may influence this association. Fourth, since the data was derived from only one college in China, it may not be representative of all Chinese college students or other populations.

## 5. Conclusions

Our findings suggest that better physical fitness, including running ability, pull-ups, standing long jump, flexibility, and vital capacity, were independently related to a lower risk of anxiety symptoms in college students. Therefore, establishing good physical fitness may be crucial for college students’ mental health, and this is also significant information in the fields of prevention medicine and health education. It is well known that poor mental health can lead young adults to social withdrawal and even suicide. In addition, young adulthood is a key period for the formation of an individual’s lifestyle and mental status in the process of growth. Considering this, the present findings could be popularized to educators, especially physical education and psychological instructors in universities, to emphasize the importance of physical exercise to students and to develop efficient educational methods to improve students’ physical fitness and mental health. In addition, it is important to encourage students to receive psychiatric support when needed. This is important for maintaining their mental health. Interventional and prospective studies are required to examine the causality of the association between physical fitness and anxiety symptoms.

## Figures and Tables

**Table 1:** Characteristics of male and female participants according to anxiety symptoms.

	Male		*p* Value ^a^	Female		*p* Value ^a^
	Without Anxiety	Anxiety		Without Anxiety	Anxiety	
*n*	3822	2813		2553	1929	
BMI (kg/m^2^) ^b^	22.0 (21.9, 22.1) ^c^	22.1 (22.0, 22.2)	0.163	20.4 (20.3, 20.5)	20.4 (20.3, 20.6)	0.302
Grade (%)						
Freshman	20.4	41.0	<0.001	20.4	45.6	<0.001
Sophomore	30.3	26.2	<0.001	35.1	27.0	<0.001
Junior	27.6	24.2	0.002	33.6	22.0	<0.001
Senior	21.7	8.6	<0.001	10.9	5.3	<0.001
Minority race (%)	4.1	3.7	0.523	5.2	6.5	0.069
Living expenses (%)						
Low	40.2	39.6	0.630	34.9	35.3	0.800
Medium	50.2	51.5	0.297	53.9	53.6	0.832
High	9.6	8.9	0.326	11.2	11.1	0.962
Living status (dormitory; %)	98.9	99.1	0.381	99	99.3	0.325
Nonsmoker (%)	91.3	91.6	0.657	99.5	99.5	1.000
Drinking status (%)						
Nondrinker	70.3	66.1	<0.001	95.9	94.8	0.082
Drink 1–2 times/week	26.5	30.5	<0.001	3.9	4.6	0.293
Drink > 2 times/week	3.2	3.4	0.577	0.2	0.6	0.011
PA (METs hour/week)	51.7 (50.3, 53.2)	50.7 (49.0, 52.4)	0.353	49.6 (47.7, 51.4)	51.1 (49.0, 53.2)	0.264
Sleep duration (7–8 h/day; %)	61.3	59.8	0.222	66.1	62.4	0.010
Physical fitness						
Standing long jump (cm)	231.4 (230.8, 232.0)	228.8 (228.1, 229.5)	<0.001	172.2 (171.7, 172.8)	170.3 (169.6, 170.9)	<0.001
Sit-and-reach test (cm)	12.3 (12.1, 12.5)	11.7 (11.5, 11.9)	<0.001	17.0 (16.8, 17.2)	16.8 (16.6, 17.1)	0.421
50 m sprint (Second)	7.35 (7.34, 7.37)	7.41 (7.40, 7.43)	<0.001	9.09 (9.06, 9.11)	9.15 (9.13, 9.18)	<0.001
Vital capacity (ml/kg)	65.2 (64.8, 65.5)	63.6 (63.2, 64.0)	<0.001	55.6 (55.2, 56.0)	54.0 (53.5, 54.4)	<0.001
1000 m run (Second)	255.1 (254.3, 255.9)	254.6 (253.7, 255.5)	0.396			
Pull-up (Times/minute)	4.16 (4.04, 4.28)	3.74 (3.60, 3.88)	<0.001			
800 m run (Second)				242.1 (241.3, 242.8)	244.2 (243.3, 245.0)	<0.001
Sit-up (Times/minute)				32.8 (32.5, 33.1)	32.0 (31.7, 32.4)	0.001

^a^ Obtained using *t*-test for continuous variables and χ^2^ test for proportional variables. ^b^ BMI: body mass index. PA: physical activity. ^c^ Mean (95% CI) and *n* (%) were used for continuous variables and categorical variables, respectively.

**Table 2:** Multivariate linear regression models with GAD-7 score as dependent variable.

	GAD-7 Score					
	Male			Female		
	B (95%CI)	Beta	*p*	B (95%CI)	Beta	*p*
50-m sprint (second)						
Unadjusted	0.70 (0.51, 0.89)	0.09 ^a^	<0.001	0.36 (0.18, 0.55)	0.06	<0.001
Adjusted ^b^	0.45 (0.25, 0.64)	0.06	<0.001	0.13 (−0.05, 0.31)	0.02	0.160
Sit-and-reach test (cm)						
Unadjusted	−0.04 (−0.05, −0.02)	−0.06	<0.001	−0.02 (−0.04, 0.00)	−0.03	0.066
Adjusted	−0.03 (−0.04, −0.01)	−0.05	<0.001	−0.01 (−0.03, 0.01)	−0.02	0.301
Standing long jump (cm)						
Unadjusted	−0.02 (−0.02, −0.02)	−0.10	<0.001	−0.02 (−0.03, −0.01)	0.08	<0.001
Adjusted	−0.01 (−0.02, −0.01)	−0.06	<0.001	−0.01 (−0.02, −0.00)	−0.04	0.008
Vital capacity (ml/kg)						
Unadjusted	−0.03 (−0.03, −0.02)	−0.08	<0.001	−0.03 (−0.04, −0.02)	0.10	<0.001
Adjusted	−0.01 (−0.02, −0.00)	0.04	0.008	0.00 (−0.01, 0.01)	0.00	0.826
Pull-up (times/minute)						
Unadjusted	−0.06 (−0.08, −0.04)	−0.07	<0.001			
Adjusted	−0.01 (−0.04, 0.01)	−0.01	0.293			
1000-m run (second)						
Unadjusted	0.00 (0.00, 0.00)	0.00	0.784			
Adjusted	0.00 (−0.00, 0.01)	0.01	0.440			
800-m run (second)						
Unadjusted				0.01 (0.00, 0.02)	0.06	<0.001
Adjusted				0.01 (0.01, 0.02)	0.05	0.001
Sit-up (times/minute)						
Unadjusted				−0.03 (−0.05, −0.02)	−0.06	<0.001
Adjusted				−0.01 (−0.02, 0.01)	−0.02	0.192

^a^ Results were obtained using multivariate linear regression. β: standardized regression coefficient. ^b^ Adjusted for race, grade, BMI, living status, physical activity, sleep duration, cigarette smoking, drinking habits, and living expenses.

**Table 3:** Adjusted relationship between physical fitness and anxiety symptoms in 6635 males.

	Anxiety Symptoms		
	Anxiety/All	Unadjusted Model	Adjusted Model ^a^
50-m sprint (second)			
Fast	1052/2724	1	1
Medium	756/1754	1.20 (1.07, 1.36) ^b^	1.12 (0.98, 1.27)
Slow	1005/2157	1.39 (1.24, 1.56)	1.24 (1.09, 1.40)
*p* for linear trend ^c^		<0.001	0.001
1000-m run (second)			
Fast	982/2258	1	1
Medium	905/2183	0.92 (0.82, 1.04)	1.01 (0.89, 1/14)
Slow	926/2194	0.95 (0.84, 1.07)	0.97 (0.85, 1.10)
*p* for linear trend		0.381	0.607
Standing long jump (cm)			
Short	1065/2281	1	1
Medium	874/2176	0.77 (0.68, 0.86)	0.84 (0.74, 0.95)
Long	874/2178	0.77 (0.68, 0.86)	0.91 (0.80, 1.03)
*p* for linear trend		<0.001	0.125
Sit-and-reach test (cm)			
Short	1017/2221	1	1
Medium	906/2205	0.83 (0.73, 0.93)	0.89 (0.79, 1.01)
Long	890/2209	0.80 (0.71, 0.90)	0.84 (0.74, 0.95)
*p* for linear trend		<0.001	0.006
Pull-up (times/minute)			
Low	566/1114	1	1
Medium	1325/3126	0.71 (0.62, 0.82)	1.28 (1.08, 1.51)
High	922/2395	0.61 (0.53, 0.70)	1.04 (0.92, 1.17)
*p* for linear trend		<0.001	0.011
Vital capacity (ml/kg)			
Low	1028/2211	1	1
Medium	950/2212	0.87 (0.77, 0.98)	0.98 (0.86, 1.13)
High	835/2212	0.70 (0.62, 0.79)	0.82 (0.70, 0.95)
*p* for linear trend		<0.001	0.007

^a^ Adjusted for race, grade, BMI, living status, physical activity, sleep duration, cigarette smoking, drinking habits, and living expenses. ^b^ Variables are expressed as odds ratios (95% CI). ^c^ Obtained using multivariate logistic regression analysis.

**Table 4:** Adjusted relationship between physical fitness and anxiety symptoms in 4482 females.

	Anxiety Symptoms		
	Anxiety/All	Unadjusted Model	Adjusted Model ^a^
50-m sprint (second)			
Fast	697/1739	1	1
Medium	557/1282	1.15 (0.99, 1.33) ^b^	1.09 (0.94, 1.27)
Slow	675/1461	1.28 (1.12, 1.48)	1.12 (0.97,1.30)
*p* for linear trend ^c^		<0.001	0.124
800-m run (second)			
Fast	622/1519	1	1
Medium	674/1556	1.10 (0.96, 1.27)	1.17 (1.01, 1.36)
Slow	633/1407	1.18 (1.02, 1.37)	1.22 (1.04, 1.43)
*p* for linear trend		0.027	0.012
Standing long jump (cm)			
Short	835/1790	1	1
Medium	507/1203	0.83 (0.72, 0.97)	0.91 (0.78, 1.07)
Long	587/1489	0.74 (0.65, 0.86)	0.84 (0.73, 0.97)
*p* for linear trend		<0.001	0.021
Sit-and-reach test (cm)			
Short	728/1691	1	1
Medium	598/1343	1.06 (0.92, 1.23)	1.11 (0.96, 1.30)
Long	845/603	0.94 (0.82, 1.09)	0.99 (0.85, 1.15)
*p* for linear trend		0.461	0.928
Sit-up (times/minute)			
Low	878/1918	1	1
Medium	503/1185	0.87 (0.76, 1.01)	0.99 (0.85, 1.15)
High	548/1379	0.78 (0.68, 0.90)	0.91 (0.78, 1.06)
*p* for linear trend		<0.001	0.226
Vital capacity (ml/kg)			
Low	721/1492	1	1
Medium	636/1495	0.79 (0.69, 0.92)	0.96 (0.82, 1.13)
High	572/1495	0.66 (0.57, 0.77)	0.96 (0.81, 1.15)
*p* for linear trend		<0.001	0.677

^a^ Adjusted for race, grade, BMI, living status, physical activity, sleep duration, cigarette smoking, drinking habits, and living expenses. ^b^ Variables are expressed as odds ratios (95% CI). ^c^ Obtained using multivariate logistic regression analysis.

## Data Availability

The datasets used to support the findings of this study are available from the corresponding author upon reasonable request.
